# Faster growth with shorter antigens can explain a VSG hierarchy during African trypanosome infections: a feint attack by parasites

**DOI:** 10.1038/s41598-018-29296-8

**Published:** 2018-07-19

**Authors:** Dianbo Liu, Luca Albergante, T. J. Newman, David Horn

**Affiliations:** 1 School of Life Sciences, University of Dundee, Dundee, DD1 5EH UK; 2 Institut Curie, PLS Research University, Mines Paris Tech, Inserm U900, F-75005 Paris, France; 3 The Broad Institute of MIT and Harvard, 415 Main Street, Cambridge, MA 02142 USA; 4 Computer Science and Artificial Intelligence Lab, Massachusetts Institute of Technology, 32 Vassar St, Cambridge, MA 02139 USA; 5 Solaravus, PO Box 29476, Cupar, KY15 9AS UK; 6 Wellcome Trust Centre for Anti-Infectives Research, School of Life Sciences, University of Dundee, Dundee, DD1 5EH UK

## Abstract

The parasitic African trypanosome, *Trypanosoma brucei*, evades the adaptive host immune response by a process of antigenic variation that involves the clonal switching of variant surface glycoproteins (VSGs). The VSGs that come to dominate *in vivo* during an infection are not entirely random, but display a hierarchical order. How this arises is not fully understood. Combining available genetic data with mathematical modelling, we report a VSG-length-dependent hierarchical timing of clonal VSG dominance in a mouse model, consistent with an inverse correlation between VSG length and trypanosome growth-rate. Our analyses indicate that, among parasites switching to new VSGs, those expressing shorter VSGs preferentially accumulate to a detectable level that is sufficient to trigger a targeted immune response. This may be due to the increased metabolic cost of producing longer VSGs. Subsequent elimination of faster-growing parasites then allows slower-growing parasites with longer VSGs to accumulate. This interaction between the host and parasite is able to explain the temporal distribution of VSGs observed *in vivo*. Thus, our findings reveal a length-dependent hierarchy that operates during *T. brucei* infection. This represents a ‘feint attack’ diversion tactic utilised by these persistent parasites to out-maneuver the host adaptive immune system.

## Introduction

*Trypanosoma brucei*, a species of parasitic protozoan, is transmitted by tsetse flies and is responsible for African sleeping sickness in humans and nagana in animals. The diseases result in thousands of human deaths per year and pose a serious humanitarian and economic threat to developing countries. The *T. brucei* parasites display variable surface glycoproteins (VSGs)^1–3^ which coat the cell surface. VSGs are recognised as antigens by the adaptive immune system of the host, which reacts by mounting a VSG-specific immune response. However, each *T. brucei* cell has the ability to dynamically change its specific VSG, therefore forcing the immune system to continuously adapt to the ever-changing ‘antigenic landscape’. This process of immune evasion can continue for years in a single host.

Given their importance as mediators of a successful infection, the mechanisms that control the switching and expression dynamics of genes encoding VSGs and the resulting host-parasite interactions are active fields of study. Several aspects of the dynamics of VSG expression have been elucidated. For example, it is known that only one of approximately 15 VSGs, found in specific genomic locations close to the telomeres (so-called expression sites), is active in any given cell^4^. However, many more genes (up to two thousand) are present in other genomic locations. The genes found in this archive need to be copied to the active site, through a gene-conversion mechanism, to be expressed^5^. In this *VSG* gene repertoire of *T. brucei*, less than 15% of the genes are intact. The vast majority are pseudogenes or gene fragments^6,7^ that, nevertheless, can come together to form functional mosaics^8,9^.

Several previous efforts have been made to use mathematical models to understand the complex population dynamics of parasites and their interactions with the host immune system. In the case of *T. brucei*, these models considered various factors that potentially underlie population behaviour, especially the semi-predictable order of appearance of VSGs *in vivo*. The range of mechanisms considered includes different probabilities of activation of VSGs, differential switching rates of variants^10,11^, density-dependent differentiation from the replicative (slender) form to the non-replicative (stumpy) form of the parasites^12,13^, clustering of groups of variants^12^, and selection by the host immune system^14^. These models and parameters may also apply to other parasites, such as *Plasmodium falciparum*^11,15^. For these latter parasites, *in vitro* analysis reveals a highly structured switching pattern^16^, while mathematical models predict that conflicting immune responses can prolong the infection^17^. Here, we propose VSG length as a fundamental principle underlying antigenic ordering and find that our hypothesis is supported by *in vivo* experimental data.

Prior observations indicate that parasites expressing VSG-2 (aka. 221), which is one of the shortest VSGs in the genome (12^th^ percentile)^18^, consistently appears in the first-relapse populations *in vivo*^19^ and often outcompetes *T. brucei* expressing other VSGs *in vitro*^4,18,19^. In addition, VSGs in *T. brucei* have a range of lengths. These facts led us to ask whether *T. brucei* clones expressing shorter VSGs could have a survival advantage, possibly due to the increased metabolic cost of producing longer VSGs.

To this end, we constructed a minimal mathematical model to study the potential effect of differing VSG lengths on parasite population dynamics. The model was tested against *in vivo* experimental data obtained from mice^9^ and was used to explore how differential VSG-switching, or impact on growth, could contribute to the survival of the parasite in an immuno-competent host. Modelling indicates that faster growth of parasites expressing shorter VSGs extends the duration of infection. This particular model also explains the hierarchy of VSG expression observed *in vivo* in mouse infections.

## Results

### Modelling VSG expression dynamics

**Figure 1:**
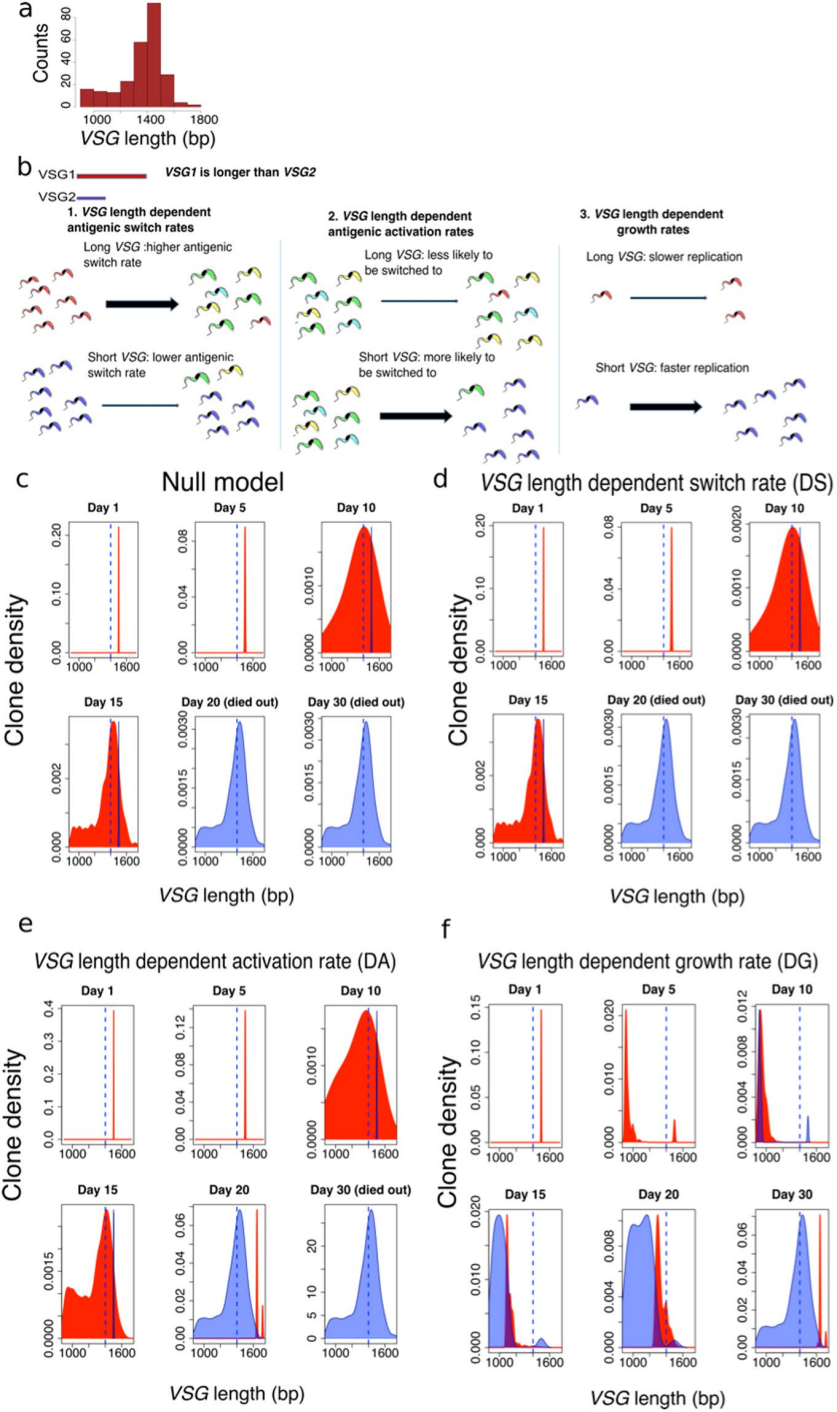
VSG length-dependent population dynamics of *T. brucei*. (**a**) Distribution of lengths for all 252 *VSGs* detected in four mouse infections^9^. (**b**) Three potential mechanisms are explored: 1) *VSG* length-dependent switch rate (DS), in which *T. brucei* with short *VSGs* switch surface antigens less frequently; 2) *VSG* length-dependent activation rate (DA), in which short *VSGs* are more likely to be activated; and 3) *VSG* length-dependent growth rate (DG), in which parasites with shorter *VSGs* replicate faster. We also considered a ‘negative control’ (null) model in which all the parasites have the same switching and replication rates, and all *VSGs* are equally likely to be activated. (**c**–**f**) The red area indicates the expressed *VSG* length distribution in a population of parasites, while the blue area reports *VSGs* that have been detected by the host adaptive immune system. Blue dashed line indicates the median length of all *VSGs* in the library. All the densities in the Figures refer to clone density (Kernel estimation).

One striking aspect of *T. brucei VSGs* is the broad distribution of lengths, despite those genes apparently having similar functions^4,18^. Figure 1a shows the distribution of *VSG* lengths from the full set of 252 expressed *VSGs* detected in mouse infections^9^. This suggests that the length of *VSGs* may have an intrinsic biological significance. Inspired by previous experimental observations that *T. brucei* variants with shorter *VSGs* often dominate *in vitro*^4,18,19^, we used a mathematical model to explore the potential effect of length variation of *VSGs* on the intra-host parasite population dynamics. In creating a simple mathematical model of *T. brucei* infection focused on the length dependence of *VSGs*, we introduce three different plausible molecular mechanisms that potentially relate the length of the active *VSG* to the behaviour of the parasite population, as shown in Fig. 1b: (1) *VSG* length-dependent switch (DS) in which *T. brucei* with shorter *VSGs* switch surface antigens less frequently. For example, this could be due to reduced occurrence of switch-inducing DNA breaks within shorter *VSGs*; (2) *VSG* length-dependent activation (DA) in which shorter *VSGs* are more likely to be activated. For example, this could be due to more efficient gene-conversion over shorter distances; and (3) *VSG* length-dependent growth (DG) in which parasites with shorter *VSGs* replicate faster. This could be due to faster *VSG* production with a lower metabolic cost. We also considered a ‘negative control’ null model in which all parasites have the same switching and replication rates, and all *VSGs* are equally likely to be activated.

Considering a population of *T. brucei* in the bloodstream of the host, we denote the density (number of parasites per ml of blood) of variant *k* by *N*_*k*_. The antigenic switching rate of each variant is also *VSG*-length-dependent and is denoted by α_*k*_ (and is restricted to the biologically plausible range of 0 to 2 × 10^−4^ switches per parasite per replication). A putative *VSG*-length-dependent antigenic activation rate of variant *k* is dictated by *Q*_*k*_, which describes the preferential switching to *VSG* of variant *k* in the population, and is normalised to unity. The intrinsic growth rate of each variant depends on its *VSG*-length. We denote by *r*_*k*_ the length-dependent net growth rate of variant *k* (which accounts for cell replication and cell death due to factors other than adaptive immune killing, and is restricted to the biologically plausible range of 0 to 4 replications per parasite per day). *I*_*k*_ denotes the rate of adaptive immune killing of variant *k* and is set to the biologically plausible value of 5 kills per parasite per day (*I*_*k*_ = 5)^12^ with the acquired immune killing threshold chosen to be equivalent to the detection of a variant when it reaches 10^2^ parasites/ml and a time delay of 5 days (associated with T-cell priming and expansion)^20^.The specific forms of the length-dependency of *r*_*k*_, *α*_*k*_, *Q*_*k*_, *I*_*k*_ are described in detail in the Materials and Methods section.

Thus, we modelled the dynamics of *T. brucei* parasites in the bloodstream of the host using the following system of equations:

$$\frac{d{N}_{k}}{dt}={r}_{k}{N}_{k}\,\mbox{--}\,{\alpha }_{k}{N}_{k}\,\mbox{--}\,{I}_{k}{N}_{k}+{Q}_{k}\sum _{i=1}^{M}{\alpha }_{i}{N}_{i}$$

For a given value of *k* (i.e. a given variant) the left-hand-side describes the net change of *N*_*k*_ over time. The right-hand-side comprises the set of biological processes through which *N*_*k*_ can change. ${Q}_{k}{\sum }_{i=1}^{M}{\alpha }_{i}{N}_{i}$ denotes the net switching rate of the whole *T. brucei* population to a specific variant *k*, where *M* is the total number of variants. Note that in our model the immune system adapts to (i.e. recognises) *VSG* variants over time. In particular, the modelled adaptive immune system will initiate killing of a given VSG-expressing clone 5 days after it reaches a set threshold and will continue killing the clones until the infection has been cleared or the host dies (see Materials and Methods for more details). Unless stated otherwise, model infections were initiated with 1000 parasites per ml of blood expressing a single *VSG* of 1500 bp in length. The system of ordinary differential equations was numerically solved to study population dynamics of parasites with different *VSG* lengths over a 30-day timeframe.

Potential cross-reactivity of the adaptive immune system was not considered in our model due to the extensive parametrization required. Another feature absent from our model is quorum sensing, balancing proliferative slender-form and arrested stumpy-form parasites^21^, since the vast majority of natural human infections are characterised by a low level of bloodstream-parasitaemia, in which case quorum-sensing would not be expected to have a major impact on antigenic variation.

In the null model, the distribution of *VSG* lengths and the *VSG* spectrum detected by the adaptive immune system tend to follow a similar pattern, only with a time-delay for the immune response (Fig. 1c). Die-out or ‘End Time’ does vary when different growth-rates and immune killing rates are considered but varies very little when different switch-rates or immune thresholds are applied to the null model (Supplementary Figure S1a–f). Similar dynamics are observed in the length-dependent switch (DS) model (Fig. 1d). A different scenario emerges in the length-dependent activation (DA) model in which we observe an increased representation of shorter *VSGs* between 10–15 days and an infection dominated by longer *VSGs* on day 20 (Fig. 1e). This trend from shorter to longer *VSGs* is much stronger in the length-dependent growth (DG) model (Fig. 1f). In this case, the detected *VSG* spectrum is substantially different at each time-point sampled and remains one step ahead of the immune response throughout the time-course (Fig. 1f, Supplementary Figure S2a,b); the length distribution of the expressed *VSG* initially moves towards shorter antigens, but by day 15 the trend is reversed and the distribution shifts toward longer *VSGs*. Our results stand even when more complex immune system models that detect antigens in a probabilistic manner are considered, where the probability for adaptive immune system to detect a specific clone follows an exponential distribution (Supplementary Figure S2c), or when a different *VSG* library is used (Supplementary Figure S2d).

This analysis indicates that *VSG* length-dependent growth rate, and to a lesser extent, *VSG* length-dependent activation rate, would allow *T. brucei* to establish a more persistent infection. This is because the adaptive immune system is unable to generate responses that match the actual distribution of antigens expressed by the parasites, due to the inevitable lag in *VSG* detection and the associated immune response. This is reminiscent of a ‘feint attack’ in military tactics. The *T. brucei* population diverts the host-acquired immune system using variants with shorter VSGs, allowing variants with longer VSGs to emerge from below-threshold sub-populations. This strategy also works in the opposite direction initially if the inoculum comprises parasites expressing a relatively long VSG. When a combination of all three models was considered, the population dynamics were similar to what we observed using the DG model alone (Supplementary Figure S2e), indicating that a DG mechanism can dominate differential switching and activation mechanisms.

### Experimental infection data confirm our theoretical predictions

VSG-seq has been used to investigate the dynamics of *T. brucei* infection in an immune-competent mouse model over a period of several weeks^9^. VSG-seq is a variant of RNA-seq to quantitatively track many different *VSGs* and the abundance of cells expressing those *VSGs*. We used the data from this study to monitor the distribution of *VSG* lengths over time (see Materials and Methods).

**Figure 2:**
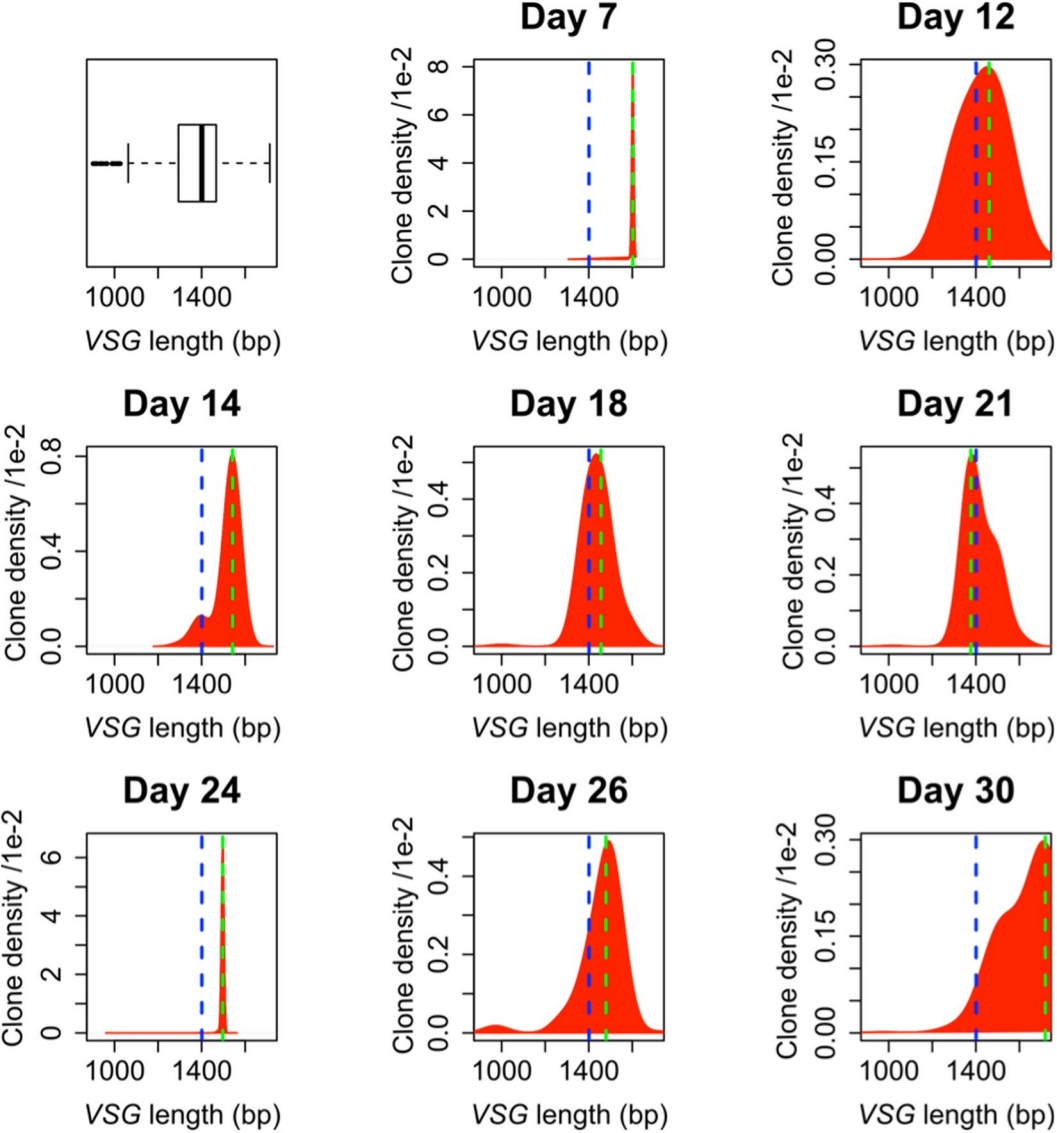
Distribution of expressed *VSG* length during *T. brucei* infection. The first panel shows the distribution of *VSG* lengths as in Fig. 1a but in box-plot format. The other panels report the distribution of *VSGs* expressed in the bloodstream of mouse number 4 in Mugnier *et al*.^9^. Note how the distribution of length shifts towards shorter *VSGs* during the initial phases of infection and then moves towards longer *VSGs* in the later phases. The blue dashed line indicates the mean length of *VSGs* in the genome and the green dashed line is the weighted mean of expressed *VSG* lengths in the population. This trend was observed in all four mice; See Supplementary Figure S3 for data from the other mice^9^.

**Figure 3:**
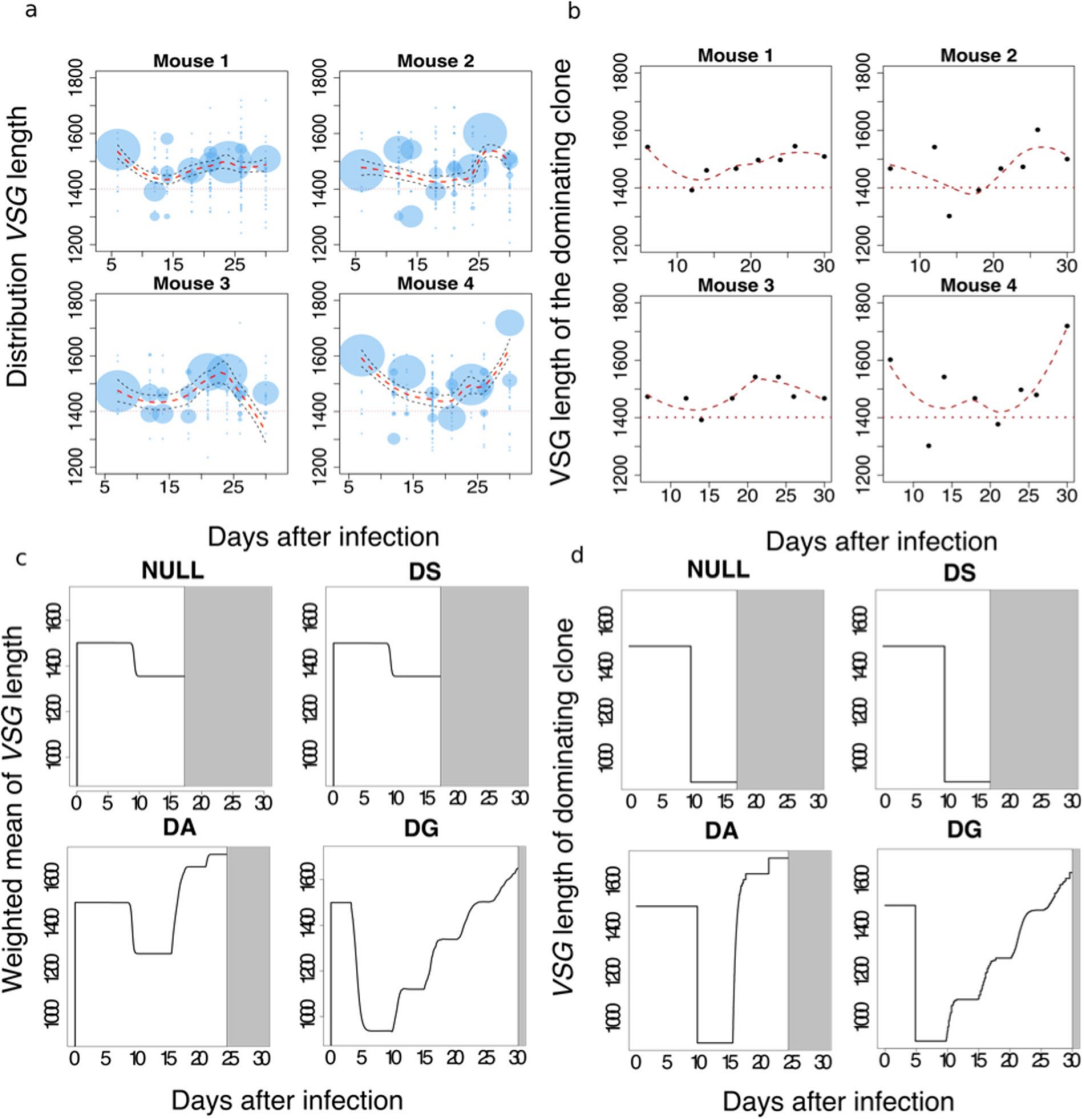
VSG length-dependent growth rate can explain *in vivo* population dynamics. (**a**) The distribution of VSG length in the detectable *T. brucei* population shows a decreasing trend followed by an increasing trend. The sizes of the circles are proportional to the percentage of VSGs of corresponding length in the population. (**b**) The VSG length of the dominating clone shows a decreasing trend followed by an increasing trend in all 4 mice. Loess regression lines are indicated in a-b. 95% confidence intervals are also indicated in a. (**c**) Simulation results show that VSG length-dependent growth rate (DG) is able to reproduce the dynamics of the weighted mean of VSG lengths in experimental data. The grey area indicates the parasite population has died out. The *T. brucei* population survived longest under the VSG length-dependent growth (DG) mechanism. (**d**) The simulation results for the VSG length of the dominating clone in the population agrees with experimental data and simulation results from panel c.

In all four mice studied, the distribution of *VSG* lengths shifts towards shorter *VSGs* during the initial phases of infection and then shifts towards longer *VSGs* in the later phases (Fig. 2, Supplementary Figure S3). This behaviour matches the predictions of our DG model, and to a lesser extent our DA model. Figure 3 shows both the distribution of *VSG* length and the *VSG* length of the clone with the largest number of parasites detected (the ‘dominant clone’) over the period of infection. In all four mice, we observe a decrease followed by an increase in both the distribution of *VSG* length and the *VSG* length of the dominant clone (Fig. 3a,b). This behaviour is compatible with both the DA model and the DG model (Fig. 3c,d), but not with alternative versions of these models; in which *T. brucei* with shorter *VSGs* switch more frequently, where shorter *VSGs* are less likely to be activated or where parasites with shorter *VSGs* replicate slower, nor in models where these parameters are randomly assigned (Supplementary Figure S4a–f). Simulations using all the *VSGs* annotated as ‘complete’ in the genome of the Lister 427 strain^18^ gave similar results (Supplementary Figure S2d). The DG model allows the parasites to survive for longer (white areas) and is therefore the model that provides the greatest advantage to the parasite. We note, however, that *VSGs* of <1300 bp do not make a major contribution to parasitaemia at any point during the mouse infections; these are typically represented by only a single sequence at each time-point (Supplementary Figure S5). This suggests that these *VSGs* typically fail to sustain robust growth, are mis-assembled sequences or that they are expressed at only a low level in some cells in the population. Thus, the range of different-length VSGs that support growth *in vivo* may in fact be restricted between approximately 1300 and 1700 bp.

Our models above were derived using an initiating parasitaemia of 1000 parasites per ml of blood. The natural infectious dose of metacyclic trypanosomes, those cells naturally transmitted from the tsetse fly salivary gland, is thought to be variable, between 5–1000 parasites, and host-parasite dependent, however^22,23^. We, therefore, asked what impact the initiating parasitaemia would have on the DG model. Initiating this model with densities of 1, 10, 100 or 1000 parasites/ml of blood revealed similar results in terms of mean *VSG* length, dominating *VSG* length and extinction time over a 30-day infection (Supplementary Figure S6a). The mean length of metacyclic *VSGs* is not statistically different from the bulk *VSGs* analysed above (Supplementary Figure S6b). Nevertheless, we also tested the impact of the initiating *VSG* length (900–1700 bp range) in our DG model. In each case, we observed a similar increasing trend in mean *VSG* length, dominating *VSG* length and extinction time over a 30-day infection (Supplementary Figure S6c). The time taken for longer *VSGs* to be detected by the immune response is consistently longer than for short *VSGs* in both cases (Supplementary Figure S6a,c, right-hand columns). Thus, our conclusions derived from the DG model are largely independent of initiating parasitaemia or *VSG* length.

We next asked whether mosaic *VSGs*, those that are assembled from segments of intact and pseudo-genes to sustain longer-term infections^8^, are longer than the average *VSG*, as might be predicted by our DG model. Mugnier *et al*.,^9^ identified three mosaic *VSGs* of 1602+/− 141 bp in length. Hall *et al*.,^8^, however, identified 187 mosaic *VSGs* from twenty-three mouse infections. These mosaic *VSGs* were initially detected 20 days after infections began, reached a high level around 30 days after infection (Supplementary Figure S6a) and were indeed significantly longer (*P* < 1e−10) than the full cohort of 346 non-mosaic *VSGs* in the Hall *et al*.^8^ dataset (Supplementary Figure S7a,b). These observations support the view that longer *VSGs* dominate the latter persistence phase of a *T. brucei* infection.

### VSG length-dependent growth enhances *T. brucei* persistence

**Figure 4:**
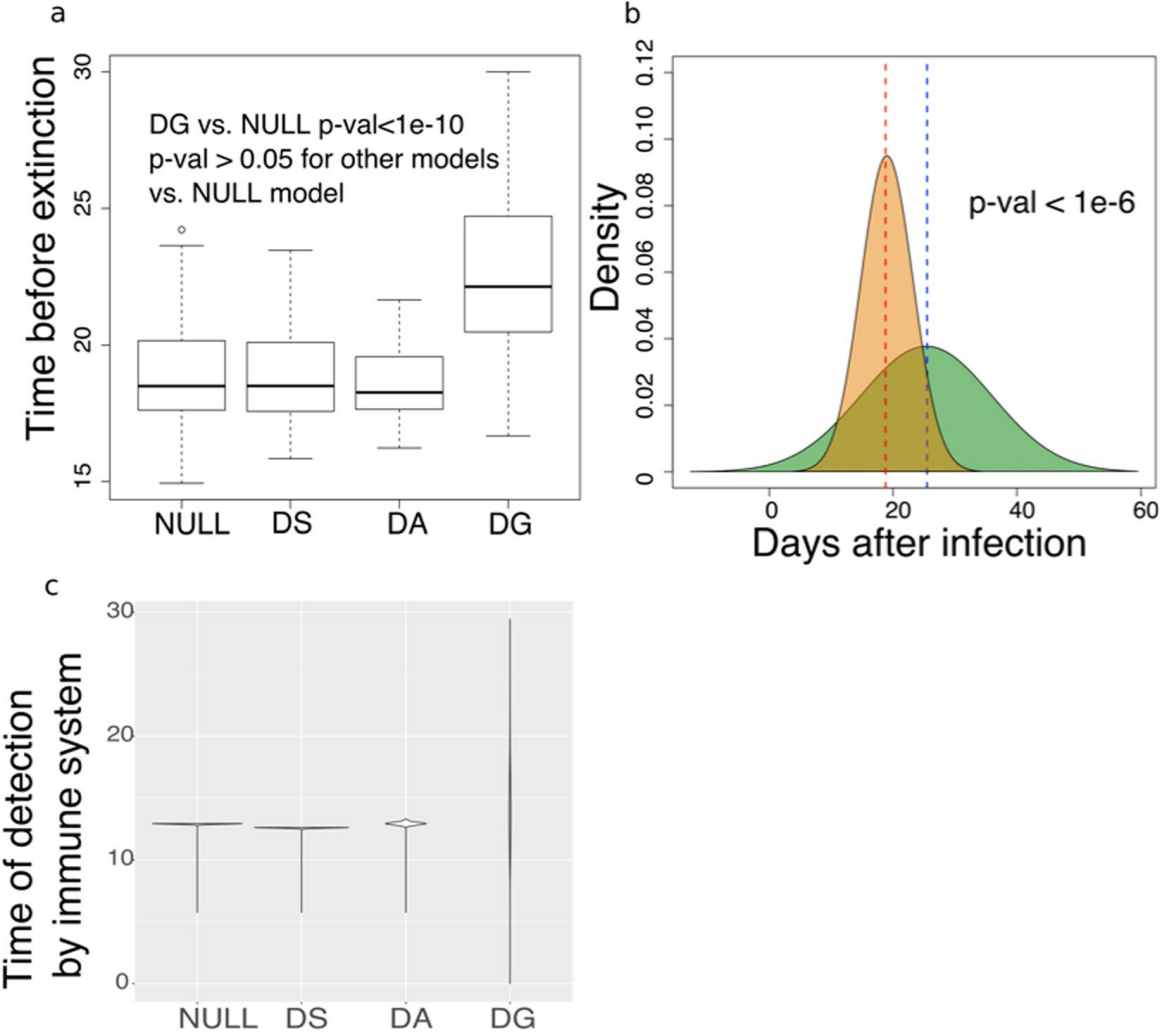
VSG length-dependent growth allows infections to persist for longer. (**a**) The distribution of times before the parasite population died out (extinction) from 500 rounds of simulation with randomly selected biologically possible parameters. Wilcoxon tests were conducted to obtain the p-values. (**b**) The distribution of time before die-out of 500 rounds of simulation of VSG length-dependent growth rate (green) vs. the null model (orange). (**c**) An example of the time taken by the adaptive immune system to detect each VSG in the population in a simulation. Length-dependent growth gives a wider distribution of immune detection times compared with other models.

Our analysis suggests that *VSG* length-dependent growth would enhance persistence. To further explore this aspect, we used our model to further investigate the time needed by the immune system to kill all parasites in an infection (the ‘extinction time’ when all the *VSGs* available are exhausted) under each of the mechanisms discussed above, excluding biological strategies such as the assembly of *VSG* mosaics. In particular, *T. brucei* survive significantly longer when the DG model is considered relative to the null model (Fig. 4a,b), while the DS and DA models fail to provide a similar advantage when considering a range of biologically possible parameters.

To provide a more precise quantification of this difference, we tracked the time taken by the adaptive immune system to detect all the *VSGs* under each different mechanism. As illustrated in Fig. 4c, the DG mechanism produces a wider distribution of immune detection times. This wide distribution is not observed in the other models (Fig. 4c). Thus, in the DG model, trypanosomes expressing shorter *VSGs*, as well as trypanosomes expressing the inoculated *VSG*, are sacrificed at the beginning of the infection. This delays the detection of others, as in a feint attack, thereby increasing the persistence of the infection.

Finally, we plotted *VSG* length against time after infection for the Hall *et al*.^8^ dataset. Information regarding population density is not available in this case but, nevertheless, there is a clear trend from short to long *VSGs* over time (Supplementary Figure S8), further supporting our hypothesis.

## Discussion

*T. brucei* carries a large library of *VSGs* in its genome, which allows a population of parasites to express a broad diversity of antigens, thus limiting the ability of the adaptive immune system to mount a curative immune response. While a diversity of antigens is necessary to evade the immune response, it is equally important that different antigens emerge in a controlled way, and in such a way to exploit the limited ability of antigen-presenting cells to identify and expose antigens present at a low concentration. This delays exhaustion of available VSGs and allows the parasite to survive longer. We have explored whether the length distribution of VSGs could be used to provide such a molecular mechanism.

Among the models considered, the hypothesis that the length of the expressed VSG causes differential growth in *T. brucei* was shown to reproduce features of *in vivo* experimental data, and provides a substantial increase in the persistence of the infection. Moreover, our model supports the idea that molecular stochastic processes can lead to a deterministically structured hierarchy of VSG length emergence times. The findings are consistent with the early appearance *in vivo*^19^ and dominance *in vitro*^4,18,19^ of the short VSG, VSG-2. VSG-length-dependent growth may also explain the 30-year-old observation of coincident multiple activations of VSG-5 (aka. 118) variants, derived through different recombination events^24^. We suggest that switch-frequency, long-debated, has a relatively minor impact on the appearance of different VSG-expressing populations *in vivo*.

VSG length-dependent growth is analogous to a ‘feint attack’ tactic of parasites that continually diverts the host immune system. Since all estimates of switching frequency to date are sufficiently high to predict the constant appearance of large numbers of switched parasites, even when parasitaemia is relatively low, the mechanism we propose does not require a transition from expressing short-to-long VSGs *per se*. Rather, a repertoire emerges constantly. The populations with shorter VSGs then grow faster and are detected earlier by the adaptive immune system. This explains why parasites expressing shorter VSGs are eliminated by the immune system first, making way for parasites expressing longer VSGs. In this scenario, the adaptive immune system lags behind the distribution of VSGs in the population, and is unable to catch up until the library of intact *VSG* genes has been exhausted. At this point, we expect that other mechanisms, such as the assembly of mosaic *VSGs*^8^ is needed to maintain the infection. The suggested mechanism provides a way for the parasite to produce a semi-predictable hierarchy of VSGs, with shorter antigens emerging early and longer antigens emerging later.

Our observations suggest a multistage, evolutionarily optimized strategy of *T. brucei*, spanning establishment, maintenance and persistence phases of infection. During the first few days of infection, a set of metacyclic *VSGs*, those initially expressed in the fly salivary gland, dominate. This stage is followed by activation of a new set of *VSGs* located in the bloodstream expression sites. Recombination, possibly dependent upon shared homology flanking the active *VSG*^25^, then allows the activation of ‘archival’ *VSGs*, which replace ‘old’ *VSGs* in expression sites. Finally, mosaic *VSGs* emerge, often assembled from gene-fragments^8,9,25^. We propose that *VSG* length-dependent growth rate plays a key role in extending the timeframe over which each of these groups of *VSGs* are effective.

Given that VSG length has a potentially key role in the interplay between parasites and host, it is reasonable to infer that this particular evolutionary pressure maintains a *T. brucei VSG* library with a wide range of lengths. Notably, it has been suggested that the recombination mechanism that underlies the generation of mosaic *VSGs* may occur within the active expression site^9^. If this is the case, longer *VSGs* dominating later stages of infections will better facilitate segmental gene conversion by providing potentially longer substrates for homologous recombination, thereby potentially enhancing the generation of mosaics^26^.

The length of VSGs may additionally affect the ability of the immune system to recognize invariant antigens on the parasite surface. For instance, haptoglobin–hemoglobin receptors mediate heme acquisition in *T. brucei* and are located within the VSG coat^27^. These receptors are recognised by the host’s innate immune system to mediate endocytosis of trypanolytic factor 1. Previous work suggested that haptoglobin–hemoglobin receptors can protrude above the VSG coat^27^. Therefore, VSG length may impact access to this receptor and other invariant receptors^27^, such as ISG65, currently used as an immunodiagnostic antigen^28^. Indeed, it may be the exposure of invariant receptors that sets a lower size limit on VSGs, since a coat comprising very short VSGs may critically expose these receptors to adaptive immune attack. It is also notable in this regard that a trend toward longer VSGs would be expected to reduce the exposure of invariant receptors. Thus, exposure of invariant proteins may not yield an effective immune response if later populations of parasites fail to similarly expose those same receptors.

How might VSG length impact parasite growth? VSG represents the most abundant mRNA and protein in the cell, with both present at approximately 10% of total cell-load^1^. Indeed, each *T. brucei* cell translates up to 80,000 VSG molecules every minute^29^. Given their abundance, the additional time required to transcribe and translate longer VSGs and the associated metabolic cost may be sufficient to reduce growth rate; translation is particularly energy intensive and VSG glycosylation will also add to the metabolic cost of producing a VSG coat. This hypothesis is also made more attractive by the observation that shorter genes produce substantially more abundant mRNAs in *T. brucei*^30^, likely facilitating the expression of shorter proteins. Consistent with this view, it has been demonstrated that diminished VSG expression in *T. brucei* triggers a cell-cycle checkpoint that persists until the cell surface is sufficiently coated by VSG^31^ and we note that differences in parasite doubling-time will have an exponential impact at the population level.

While our analyses suggest that antigen length-dependent growth rate is able to explain the observed changes in *VSG* length over time during an infection of *T. brucei* in mice, we cannot rule out the possibility that other mechanisms, such as *VSG* length-dependent antigenic activation, immunosuppression^32^ or quorum sensing^13^, which may operate in the bloodstream, in adipose tissue^33^ or in the skin^34^, may also play a role. Nevertheless, our model emphasises the important influence not only of VSG length but also the key role of the adaptive immune response on the population dynamics of *T. brucei*. Our findings highlight how VSG length may be exploited by parasites to subvert the vertebrate immune system.

## Methods

### Model

The system of ordinary differential equations in our model was numerically solved to study population dynamics of *T. brucei* parasites in the bloodstream of a host and the interplay with the host immune system during infection under each of the three hypotheses proposed.

A deterministic approach was used to simulate antigenic switching among *VSG*s and a step function was used to mimic detection via the acquired immune system of the host. In particular, the quantities described by the equation in the main text were modelled as follows:

$${r}_{k}=2(1+\frac{{l}_{k}-{l}_{0}}{500})$$

$${\alpha }_{k}={10}^{-4}(1+\frac{{l}_{k}-{l}_{0}}{500})$$

$${Q}_{k}=\frac{1}{M}(1-2\times {10}^{-3}({l}_{k}-{l}_{0}))$$

*I* is a step function: if ${N}_{k} > {\rm{immune}}\,{\rm{threshold}},{I}_{k}=5\,else\,{I}_{k}=0$.

*M* indicates the size of the *VSG* library and *I*_0_ is the median length of all *VSGs*. The immune threshold used in our study was 10^2^ parasites/ml and there was a delay of 5 days for an adaptive immune response^20^. The *VSG* library used for our analysis was provided by the authors of Mugnier *et al*.^9^. This library includes sequences of 252 *VSGs* detected in the four mice analysed. During the initial phases of the infection (1–2 weeks) the direction of the shift of the *VSG* length distribution depends on the starting *VSG* clones. Our choice of parameters gives *r*_*k*_*, α*_*k*_ and *Q*_*k*_ in biologically plausible ranges of 0–2 × 10^−4^ switches/parasites/generation (in DS model), 0 – $2\times \frac{1}{M}$ (in DA model) and 0~4 replications/parasite/day (in DG model), respectively, for *VSGs* of different lengths. In the probabilistic model, the probability for the adaptive immune system to detect a specific clone follows an exponential distribution with a rate (λ) equal to the clone density. A number is randomly sampled from the distribution, and if it is larger than $\,\frac{1}{immune\,threshold}$, then the *T*. *brucei* clone is detected by the adaptive immune system. The probabilistic model has the same scale of clone detection and same number of parameters as the deterministic model. Latin Hypercube sampling was used to study the effect on extinction time of the *T. brucei* population when parameters of the null model are varied. The parameters include growth rate (1~4 replications/parasite/day), switch rate (1–2 × 10^−4^), immune system killing rate (4–8 per parasite/day) and immune system detection threshold (10^2^–103 parasites/ml). A dummy parameter of range 0–100 is also included as a control for stochasticity. Uniform sampling was applied to all parameters.

*T. brucei* population dynamics data were obtained from published VSG-seq data^9^ and details of each *VSG* clone and mouse were provided by the authors of that study. In their work, parasites were sampled from the bloodstream of four Balb/cByJ mice. The weighted mean of *VSG* lengths is calculated as the sum of *VSG* lengths multiplied by their percentages in the population. Local polynomial regression fitting (Loess) with default settings (span = 0.75, degree = 2) was used to capture the trend of *VSG* length changes in populations.

The time before extinction of the parasite population in the host and the time of detection of each *VSG* clone by the immune system were calculated from 500 rounds of numerical integration with random parameters sampled from the ranges indicated above and based on the *VSG* distribution described above. The Wilcoxon signed-rank test was used to obtain *p*-values of the difference between the results of different models.

Codes for simulation and statistical analyses can be found at, https://github.com/kaiyuanmifen/Trypanosome.

## Electronic supplementary material

Supplementary Figures S1-S8
