# Supplementary material for: Faster growth with shorter antigens can explain a VSG hierarchy during African trypanosome infections: a feint attack by parasites
Source: Sci Rep. 2018 Jul 19;8:10922. doi: 10.1038/s41598-018-29296-8 (PMC6053454; doi:10.1038/s41598-018-29296-8)
Supplement: Supplementary file 1 — Supplementary Figures S1-S8 [file 41598_2018_29296_MOESM1_ESM.pdf]

Faster growth with shorter antigens can explain a VSG hierarchy during African trypanosome infections: a feint attack by parasites

Dianbo Liu, Luca Albergante, T. J. Newman and David Horn

Supplementary Information:

Supplementary Figures S1-8.

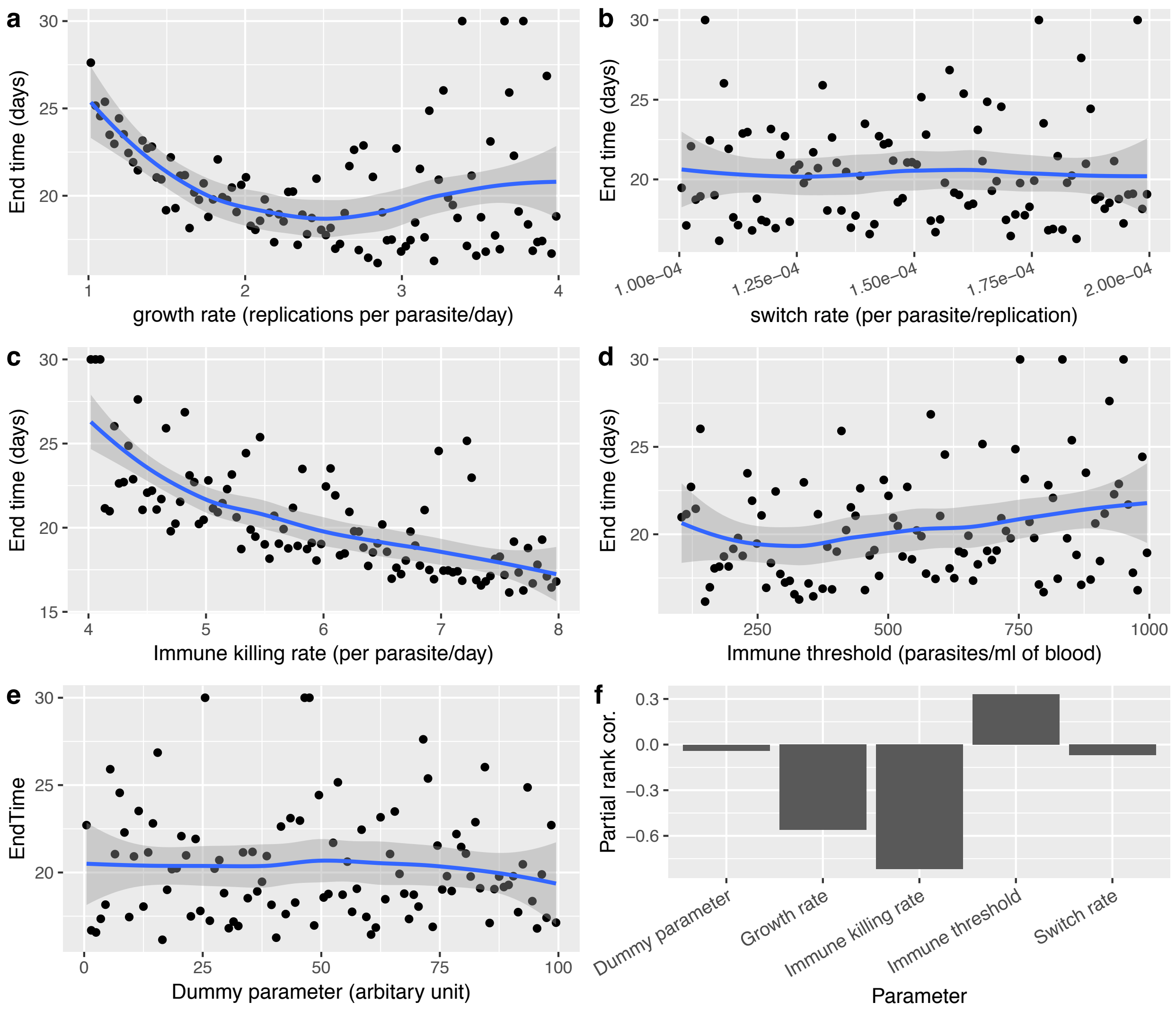

Supplementary Figure S1. Effect of varying parameters in the null model on time of extinction of the *T. brucei* population. (**a**) Growth rate vs. EndTime. (**b**) Switch rate vs. EndTime. (**c**) Immune killing rate vs. EndTime. (**d**) Immune threshold vs. EndTime. (**e**) Dummy variable vs. EndTime. Dummy variable is a random variable used to access the stochasticity of the system. (**f**) Partial rank correlation of each parameter with EndTime. When EndTime < 30 days, EndTime=ExtinctionTime.

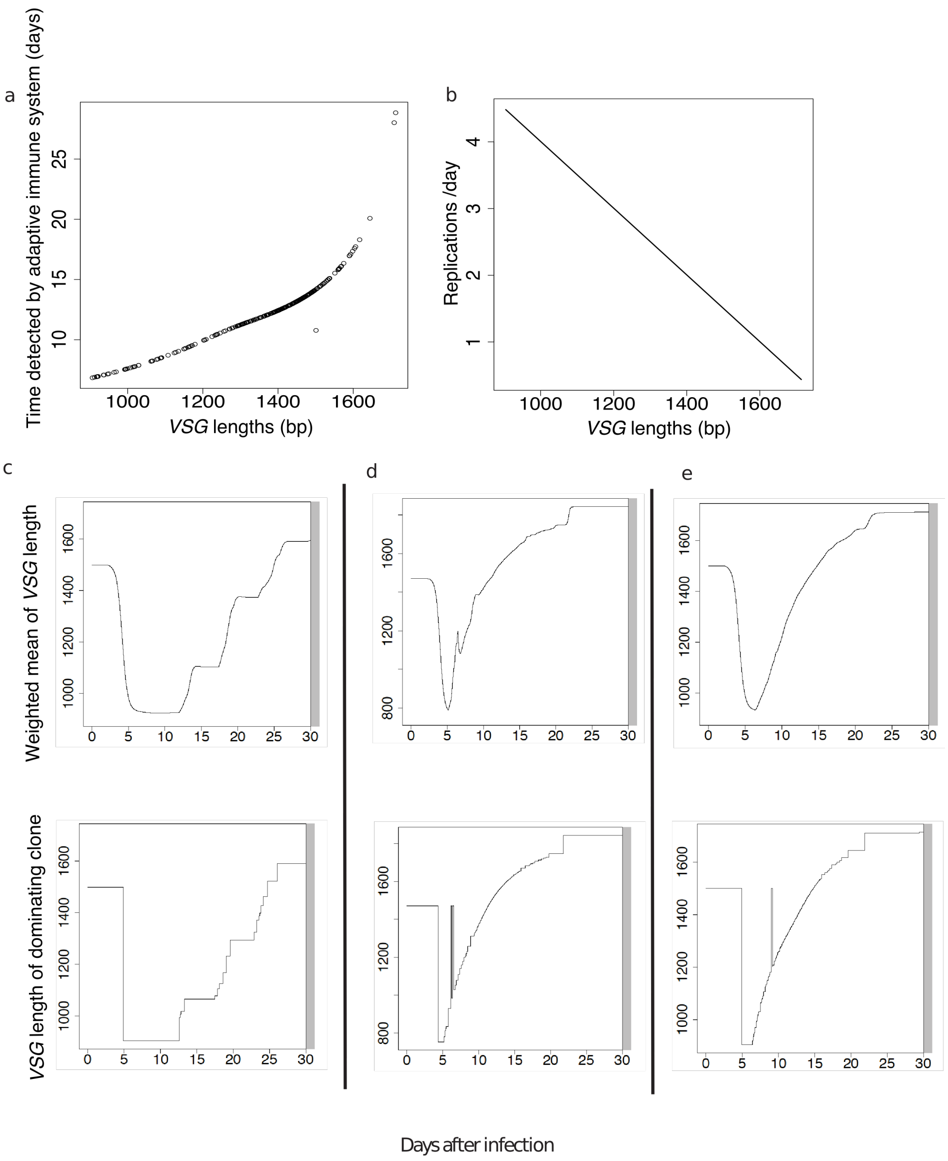

Supplementary Figure S2. Simulated infection dynamics under different model settings. (**a**) Detection times of the adaptive immune system for *VSGs* of different length under the different growth (DG) model. (**b**) Replication rate (replications/day) of *T. brucei* expressing *VSGs* of different length under the different growth (DG) model. (**c**) Weight mean of *VSG* length and *VSG* length of dominant clone under the differential growth (DG) model with an immune system that detects each *T. brucei* clone in a probabilistic manner and with an immune delay of 5 days. The method and parameter setting of the simulations are otherwise the same as shown in Figures 3c-d. (**d**) As in C above but using the *VSG* library from (18). (**e**) As in C above but combining all three models; differential switch (DS), differential activation (DA) and differential growth (DG).

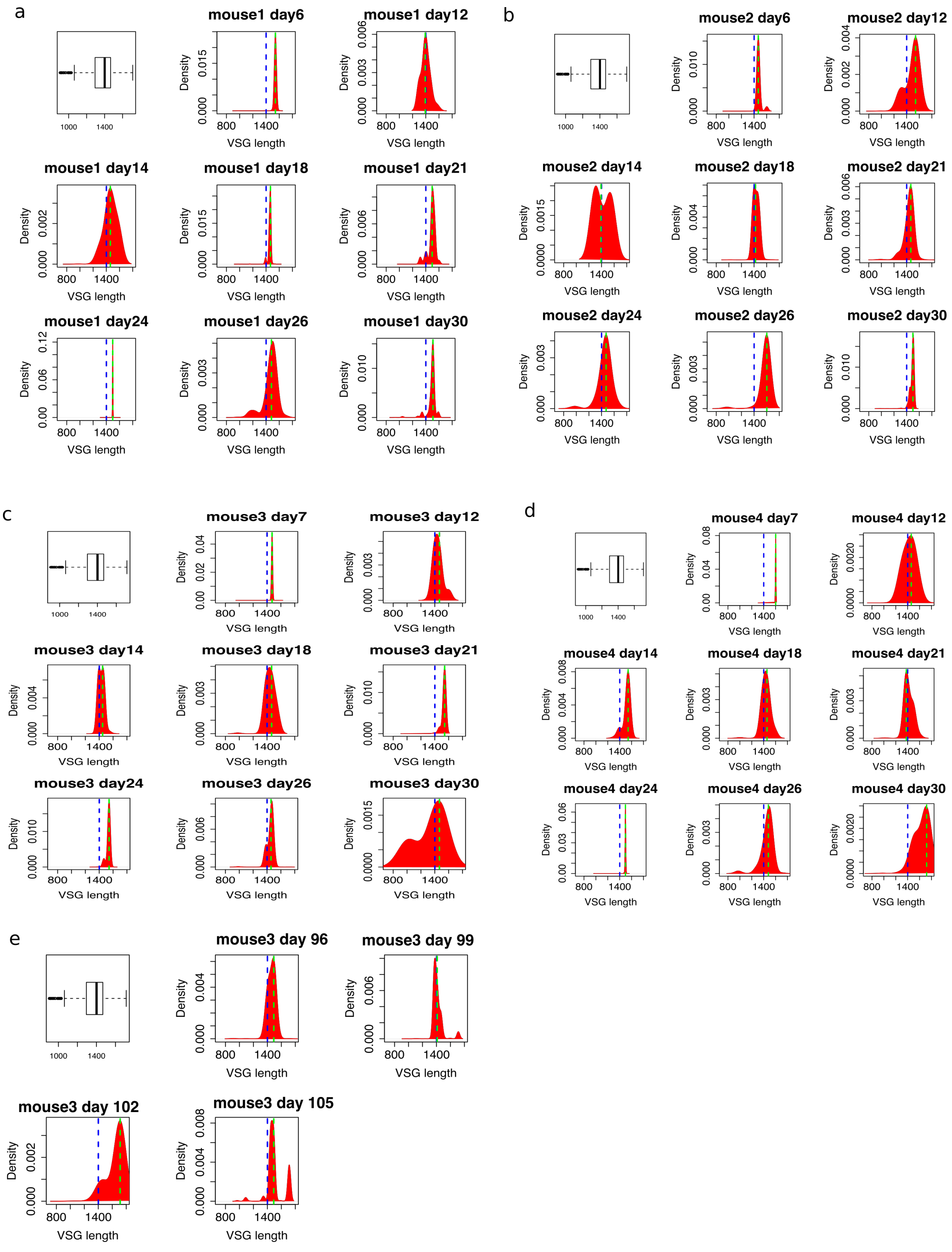

Supplementary Figure S3. Distribution of expressed *VSG* length during *T. brucei* infections. (**A-E**) Data are shown for all four mice reported by Mugnier *et al.*, (9). See Figure 2 for further details; the data for mouse 4 are also shown in Figure 2.

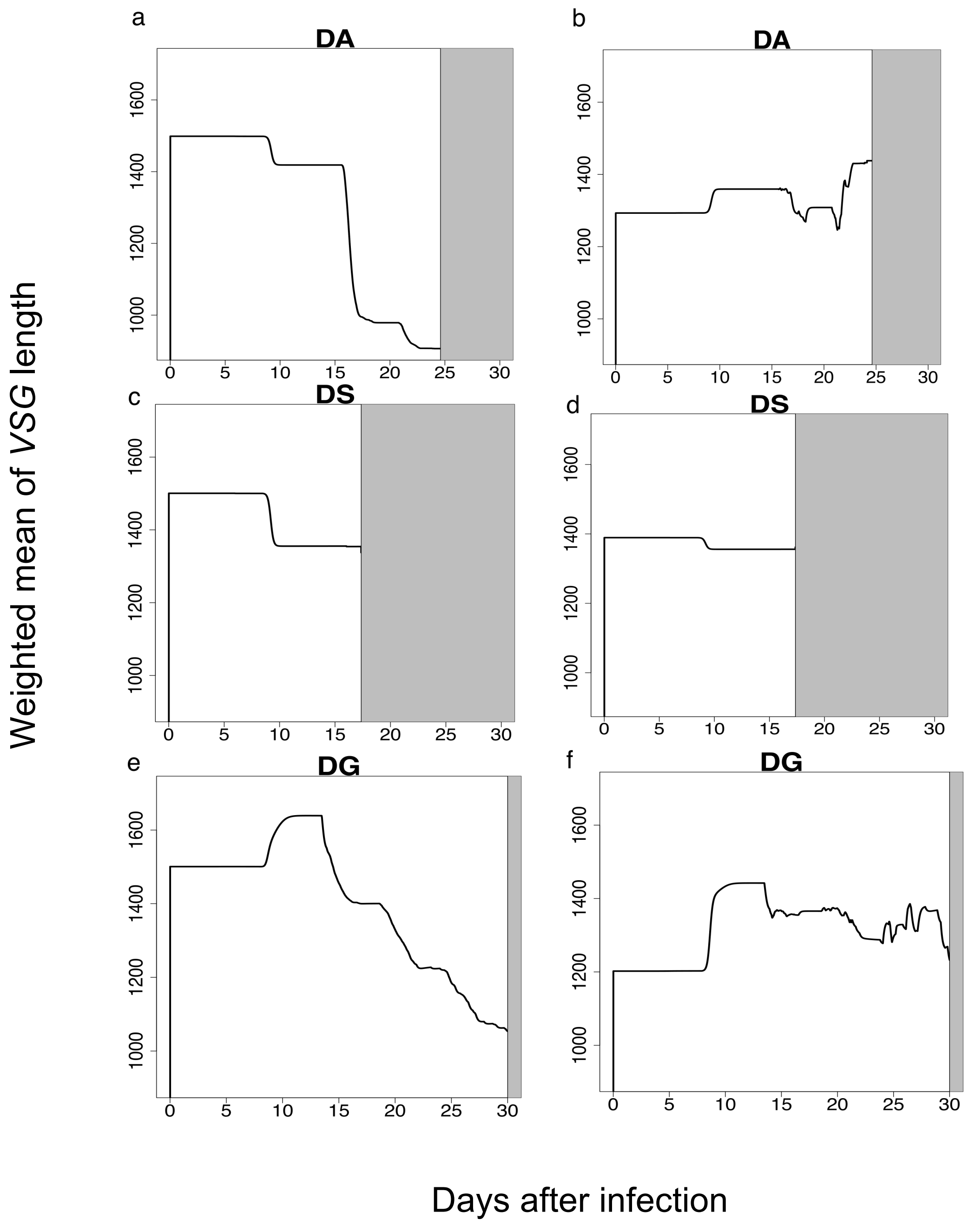

Supplementary Figure S4. Simulation results for the dynamics of the weighted mean of VSG lengths under alternative DA, DS and DG models. (**a**) DA model, but longer *VSGs* are more likely to be activated. (**b**) DA model with randomly assigned activation rates. (**c**) DS model, but shorter *VSGs* switch at a higher rate. (**d**) DS model with randomly assigned switch rates. (**e**) DG model, but clones expressing longer *VSGs* grow faster. (**f**) DG model with randomly assigned growth rate. The grey area indicates the parasite population has died out.

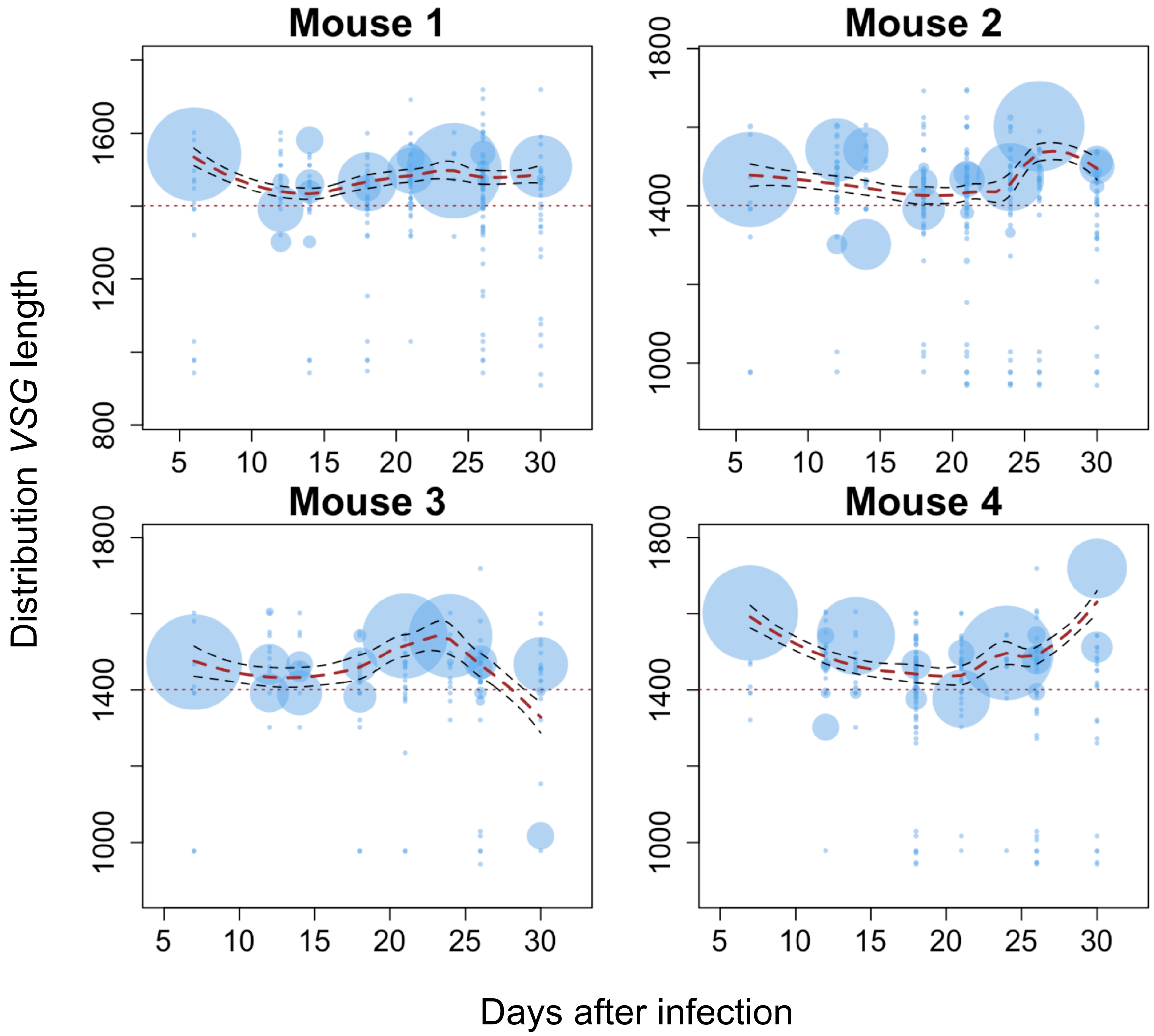

Supplementary Figure S5. VSG length-dependent growth rate can explain *in vivo* population dynamics. (**a**) All details as in Figure 3a except the vertical axes are extended to show VSG <1200 bp.

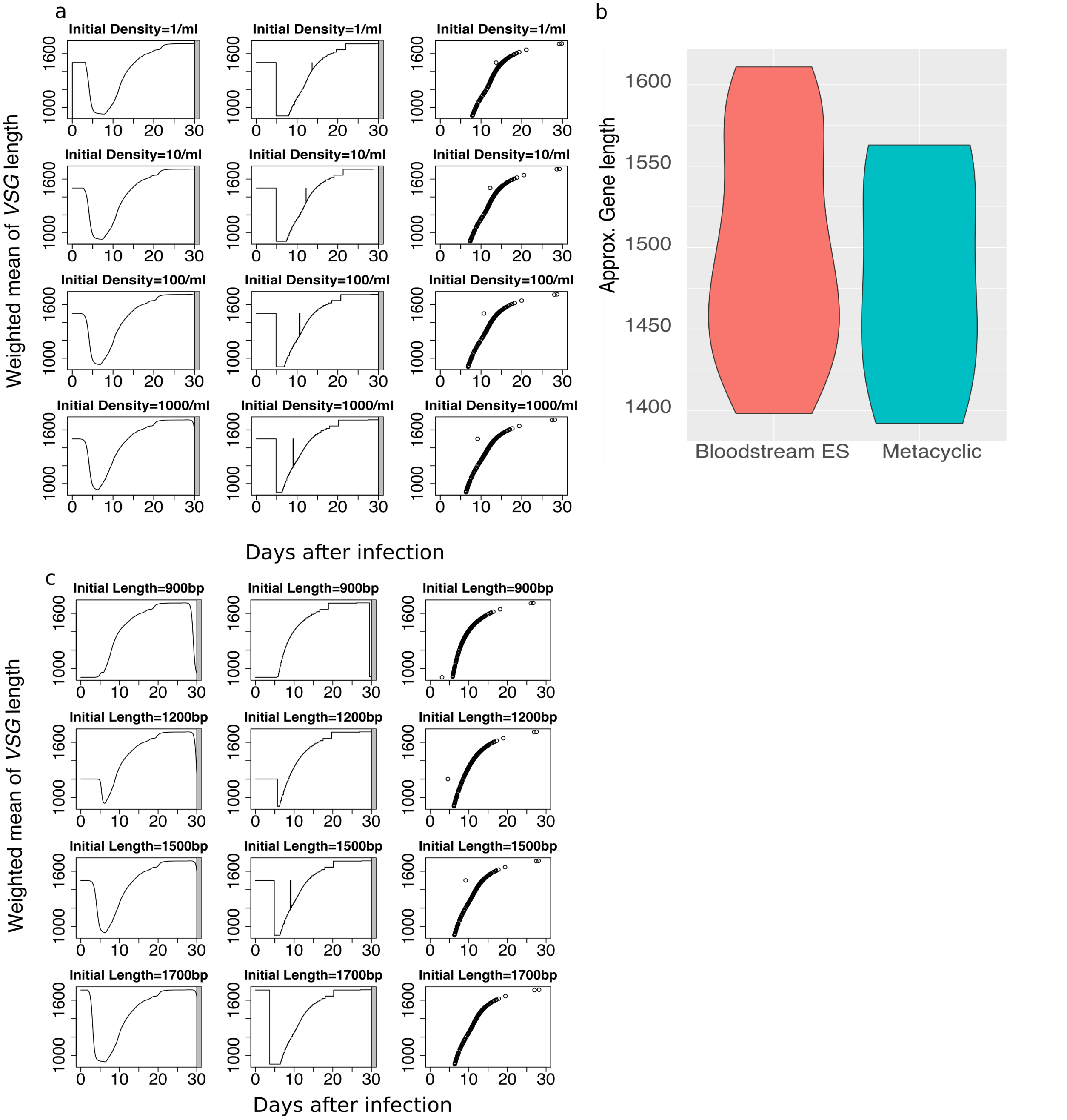

Supplementary Figure S6. VSG length-dependent population dynamics using different initial conditions. (**a**) Weight mean of VSG length (left column), VSG length of dominant clone (middle column) and length of VSG vs. time of detection by adaptive immune system (right column) under the differential growth (DG) model initiated with different parasite densities. (**b**) Metacyclic VSGs of 427 strain (n=5) at expression sites have similar lengths to their bloodstream counterparts (n=13). P value >0.8 (ks test). (**c**) Same as A above but initiated with different length VSGs. See Fig. 3c for more details.

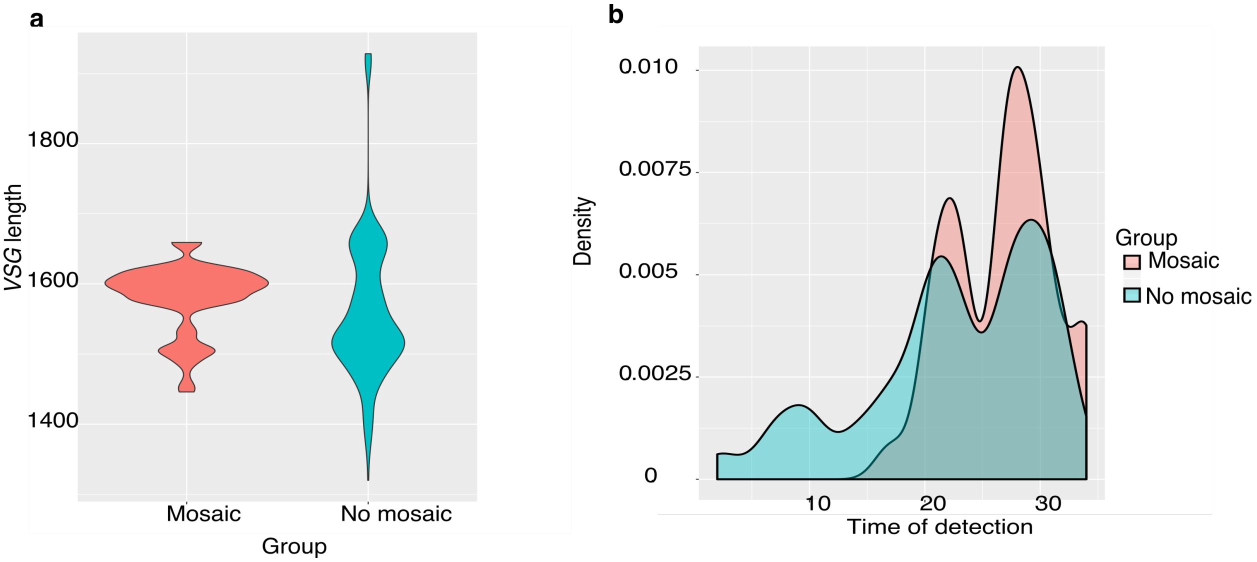

Supplementary Figure S7. Comparison between mosaic and non-mosaic *VSGs*. (**a**) Mosaic *VSGs* (n=167) are significantly longer on average (*P* value < 1e-10, ks test) than non-mosaic *VSGs* (n=346). (**b**) Mosaic *VSGs* appeared later in infections compared with non-mosaic *VSGs* (*P* value < 1e-10, ks test); these increase around day 20 and peak around day 30 after infection (8).

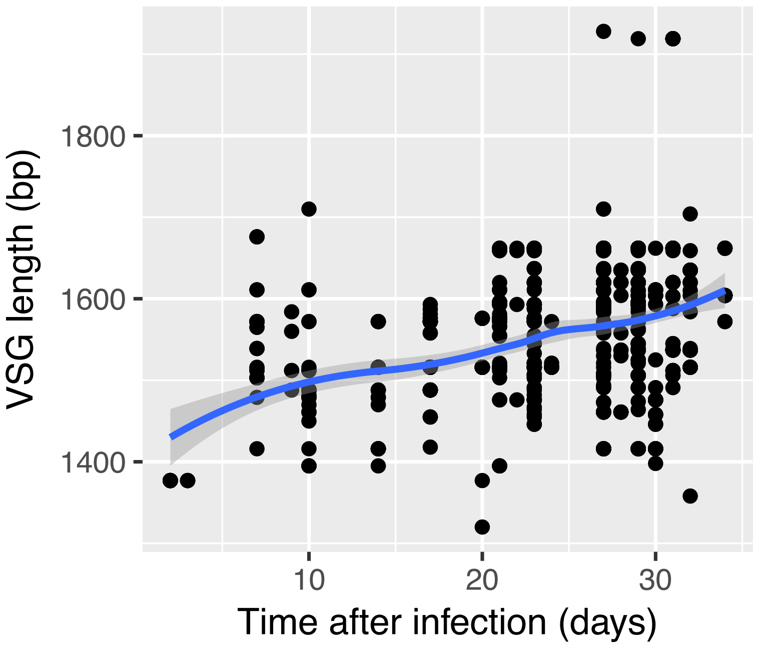

Supplementary Figure S8. Lengths of detected *VSGs* vs. days after infection for the Hall *et al*. [8] dataset, aggregating all 24 infection experiments. Each data point represents a *T. brucei* clone but without population density information (therefore not weighted). A loess line is fitted and 95% confidence interval is calculated (grey band).
